# The Beard Question

**Published:** 1861-06

**Authors:** 


The Beard Question.—*Gentlemen*:—Your article in the last number reminded me of one I wrote for a newspaper a few years ago, when the "beard question" was being actively discussed. I retained a copy of it, and, if you are willing, I should like it republished in a Medical Journal, because it points out a pathological and anatomical analogy, which, however well known, is certainly not often alluded to by writers on the subject. Yours, truly, Wm. Hunt,

*December 3d*, 1860. 431 Arch street.

"Messrs. Editors:—In the many newspaper discussions upon the beard question, I have, as yet, seen no adequate answer to the oft-repeated argument of the opposers of the appendage—'Woman has no beard, and why should man, who professes to be the stronger, need such a protection, if protection it really be?' 'Women are not intended for the exposed occupations of men,' is the general reply. Now, it strikes me that this does not at all meet the case, and I propose to give a more complete answer to this apparent poser, by showing that there is a most beautiful unity of design in the structure of the neck and face of the male and female, and, by analogy, to maintain that the beard is not only a useful protection, but an important element of manly beauty.

"The female, as a rule, is distinguished from the male by rotundity, or evenness of outline, and this, it is admitted by all, is an essential characteristic of the abstract idea of beauty. This peculiarity of form is brought about by her having, in addition to a more delicate skeleton framework, fine layers of fat immediately beneath the skin, and this fat is particularly abundant about the bust, the neck, and the face—parts that are exposed with such apparent impunity. Why impunity? Because fat is a negative substance in its physical properties, one of the best non-conductors, and hence a powerful preserver of animal heat, and, in the parts we have mentioned, a great protector of very important organs, as the summits of the lungs, the main air passages, the great blood-vessels, etc.

"So much for the female; now for the 'lord of creation.' My object is to show that the same is virtually the case with him; that what exists, as far as physical properties are concerned, *beneath* the skin in the female exists, or should exist, *upon* it in the male. He is distinguished by sharpness of outline; his skin is applied much more closely to the underlying parts. He has a much rougher skeleton, and we can more readily define the position of internal organs from external appearances in him. Hence, he protects himself by appropriate clothing to a much greater extent than his fair help meet, who, I am sorry to say, is too apt erringly to presume upon her natural advantages. Picture to yourself fifty gentlemen at an evening party with bare necks and shoulders. Fifty chills and fifty pleurisies would most probably be the result. But for the protection of parts that are necessarily exposed—the face and upper part of the neck—what is provided? Most certainly the beard! Hair, like fat, is a non-conductor, and therefore a preserver of animal heat: and, also, by its pliability, it fills up the hollows of the countenance, obviates lankness of visage, and thus contributes to that evenness of outline which we have seen is an essential element of beauty. I know it will be said that there are many fat men and many lean women. But neither of these are types of their class; besides, the former will generally be found to have smooth faces, and the latter have no more business to go in fashionable full dress, or rather undress, than men, because they not only display no beauty, but, by violating immutable laws, they too frequently contract obstinate and often incurable disease."—*Med. and Surg. Reporter.*

